# Indirect *in vitro* Regeneration of the Medicinal Plant, *Aspilia africana*, and Histological Assessment at Different Developmental Stages

**DOI:** 10.3389/fpls.2021.797721

**Published:** 2021-12-17

**Authors:** Denis Okello, Sungyu Yang, Richard Komakech, Yuseong Chung, Endang Rahmat, Roggers Gang, Francis Omujal, Alice V. Lamwaka, Youngmin Kang

**Affiliations:** ^1^Herbal Medicine Resources Research Center, Korea Institute of Oriental Medicine (KIOM), Naju-si, South Korea; ^2^Korean Convergence Medicine Major, University of Science and Technology (UST), Naju-si, South Korea; ^3^Natural Chemotherapeutics Research Institute (NCRI), Ministry of Health, Kampala, Uganda; ^4^National Semi-Arid Resources Research Institute (NaSARRI), Soroti, Uganda; ^5^Department of Pharmacy, Faculty of Medicine, Gulu University, Gulu, Uganda

**Keywords:** anatomical assessment, callus, *in vitro* propagation, organogenesis, micropropagation, *Aspilia africana*

## Abstract

The medicinal plant, *Aspilia africana*, has been traditionally used in several African countries to treat many diseases such as tuberculosis, cough, inflammation, malaria, osteoporosis, and diabetes. In this study, we developed a protocol for *in vitro* propagation of *A. africana* using indirect shoot organogenesis from leaf and root explants of *in vitro*-grown seedlings and assessed the tissues at different developmental stages. The highest callus induction (91.9 ± 2.96%) from leaf explants was in the Murashige and Skoog (MS) medium augmented with 1.0 mg/L 6-Benzylaminopurine (BAP) and 1.0 mg/L 2,4-dichlorophenoxyacetic acid (2,4-D) while from root explants, the highest callus induction (92.6 ± 2.80%) was in the same plant tissue culture medium augmented with 0.5 mg/L BAP and 1.0 mg/L 2,4-D. The best shoot regeneration capacity from leaf-derived calli (i.e., 80.0 ± 6.23% regeneration percentage and 12.0 ± 6.23 shoots per callus) was obtained in medium augmented with 1.0 mg/L BAP and 0.05 mg/L α-Naphthaleneacetic acid (NAA); the best regeneration capacity for root-derived calli (i.e., 86.7 ± 6.24% shoot regeneration percentage and 14.7 ± 1.11 shoots per callus) was obtained in the MS medium augmented with 1.0 mg/L BAP, 0.05 mg/L NAA, and 0.1 mg/L Thidiazuron (TDZ). Regenerated plantlets developed a robust root system in 1/2 MS medium augmented with 0.1 mg/L NAA and had a survival rate of 93.6% at acclimatization. The *in vitro* regenerated stem tissue was fully differentiated, while the young leaf tissue consisted of largely unorganized and poorly differentiated cells with large intercellular airspaces typical of *in vitro* leaf tissues. Our study established a protocol for the indirect regeneration of *A. africana* and offers a basis for its domestication, large-scale multiplication, and germplasm preservation. To the best of our knowledge, this is the first study to develop an indirect regeneration protocol for *A. africana* and conduct anatomical assessment through the different stages of development from callus to a fully developed plantlet.

## Introduction

The plant species *Aspilia africana* (Pers.) C. D. Adams, also known as the hemorrhage plant or wild sunflower, has been used for several centuries to treat a wide range of health conditions across Africa (Ajeigbe et al., 2014; Okello et al., 2020). Different parts of the *A. africana* plant have been used to treat tuberculosis, cough, inflammatory conditions, malaria, osteoporosis, diabetes, rheumatic pain, stomach ache, measles, ear infections, gastric ulcers, diarrhea, sores, febrile headaches, wounds, and gonorrhea, and those have also been used as a contraceptive (Okoli et al., 2007; Eweka and Eweka, 2008; Komakech et al., 2019; Okello and Kang, 2019). The plant is also known to possess potent anticancer activity (Niyonizigiye et al., 2020). The efficacy of *A. africana* in treating many diseases and managing a range of health conditions is attributed to the abundance of secondary metabolites such as flavonoids, alkaloids, saponins, phenolic compounds, and tannins in the plant (Okoli et al., 2007; Ajeigbe et al., 2014; Komakech et al., 2019).

Medicinal plants are a vital source for the discovery of novel drugs (Chen et al., 2016; Okello and Kang, 2019). Over 80% of the human population in less developed countries is fully dependent on herbal remedies for primary healthcare, and even in developed countries, one-quarter of prescribed medicines are derived from wild medicinal plants (Chen et al., 2016; Kang et al., 2018). The wild populations of medicinal plants are being increasingly threatened, mainly due to overutilization and natural habitat loss owing to the high demand of the increasing human population (Chen et al., 2016). *A. africana* although still harvested from the wild can become threatened and yet it is a valuable medicinal plant across many communities (Okoli et al., 2007; Eweka and Eweka, 2008; Okello and Kang, 2019; Niyonizigiye et al., 2020). In Cameroon, *A. africana* is the most prominent medicinal plant used to treat wounds (Simbo, 2010) while in the Eastern African country of Uganda, it is ranked as one of the top plants used to treat malaria (Okello and Kang, 2019). Although indigenous to East Africa, *A. africana* occurs in forest zones within tropical Africa regions and the savanna (Komakech et al., 2019; Okello et al., 2020). The depletion of forests in Nigeria due to anthropogenic activities including the expansion of infrastructure and agriculture has threatened the wild populations of medicinal plants such as *A. africana* (Obata and Aigbokhan, 2012). Obata and Aigbokhan (2012) emphasized that conservation strategies should be implemented for these plants to prevent the loss of these valuable resources. In a recent study, the germination rate of *A. africana* in different commercial soils was reported to be very low (<20%) in all soil categories that were investigated (Okello et al., 2021b).

Micropropagation has been used effectively for a more rapid increase in plant population than is possible with conventional propagation methods (Das et al., 2020). *In vitro* propagation of medicinal plants is essential to meet both the need for their conservation and the need to supply quality stocks to the pharmaceutical industry (Nilanthi and Yang, 2014; Komakech et al., 2020). Continuous harvests of wild medicinal plants, such as *A. africana*, without considering the domestication of these plants are a great threat to their existence (Kamatenesi et al., 2011). Kamatenesi et al. (2011) further stressed that environmental degradation, agricultural activities, and overexploitation may also lead to the eventual loss of such vital plant species. It is important to note that while *A. africana* from the wild is widely used for the treatment of diseases, its seed germination rate is very low (Okello et al., 2021b). Although there are a few studies on *in vitro* propagation of other *Aspilia* species such as *Aspilia mossambicensis* (Norton et al., 1993), there is only one study on *in vitro* propagation of *A. africana* (Okello et al., 2021c), yet it is a valuable medicinal plant of pharmaceutical interest (Komakech et al., 2019). The advantage of regenerating medicinal plants through callus is that aside from rapid multiplication of the plant, a protocol for callus induction would be established. Callus cultures have wide applications with commercial potential including secondary metabolite production for therapeutic uses and producing therapeutic antibodies along with other recombinant proteins (Efferth, 2019). The calli might possess potent biological efficacy and could be manipulated further, for example, through elicitation and metabolic engineering to upscale the secondary metabolite contents, thus, promoting its large-scale pharmaceutical use and maximizing medicinal benefits from the plant (Karwasara et al., 2010; Nandagopal et al., 2018; Efferth, 2019).

In this study, we sought to develop an indirect micropropagation method for *A. africana* using calli generated from root and leaf explants of *in vitro*-grown seedlings. Due to the economic and medicinal values of the plant, developing a micropropagation protocol for *A. africana* would greatly offset pressures on the natural populations, thus making a vital contribution to its conservation. With the proven biological efficacy of the *in vitro* regenerated *A. africana* tissues such as the callus and juvenile roots (Okello et al., 2021a), these *in vitro* regenerated tissues could offer an alternative sustainable source of raw materials to the pharmaceutical industry. Furthermore, in this study, we performed a histological assessment of the tissues during different stages of development from callus through shoot organogenesis to fully developed plantlets. To the best of our knowledge, this is the first histological study of *A. africana* through its different stages of development. Previous histological studies of *A. africana* have focused on the microanatomical features of mature plant leaf and stem features (Mabel et al., 2014; Ekeke and Mensah, 2015).

## Materials and Methods

### Seed Preparation and Germination

Seeds of *A. africana* that had been randomly collected from mature and healthy plants from Gulu, Uganda, were provided by the Natural Chemotherapeutics Research Institute, Uganda. A voucher specimen (number KYM-KIOM-2021-1) was deposited at the Korean Herbarium of Standard Herbal Resources (Index Herbarium code: KIOM) at the Korea Institute of Oriental Medicine (KIOM), Herbal Medicine Resources Research Center, Republic of South Korea. The seeds were washed thoroughly with flowing tap water and distilled water and then surface-sterilized in ethanol (70% [v/v] for 2 min) and sodium hypochlorite (5% [v/v] for 3 min), followed by four rinses with double-distilled autoclaved water. The sterilized *A. africana* seeds thereafter were inoculated in a medium containing the Murashige and Skoog (1962) (MS) mineral solution (~70 ml) in 100 × 25 mm crystal-grade polystyrene Petri dishes (Fisherbrand™). All plant media for our study contained MS vitamins and were augmented with sucrose (3%), gelled with Gelrite (3 g/L), autoclaved for about 20 min at 121°C, and adjusted at pH 5.8. The *A. africana* seeds in cultures were kept in darkness at 25 ± 2°C until they germinated, then were exposed to a 16-h photoperiod, and maintained at 70% relative humidity. Lighting was provided by cool white fluorescent tubes (OSRAM DULUX L 55W/865, FPL 55EX-D, South Korea) with an intensity of 33.73 μmol/m^2^/s. After the germination of *A. africana* seeds, i.e., 3 weeks after inoculation, the seedlings were moved to fresh medium (same composition) in 125 × 110 mm culture vessels for growth for a further 5 weeks, after which the young leaves and roots were excised and used for the induction of callus.

### Induction of Callus

The explants (leaves and roots) were obtained from *in vitro* germinated seeds in sterile environments; therefore, they were simply rinsed (three times) with autoclaved distilled water. The leaves (excluding leaf margins) were cut into segments (0.4–0.6 cm wide and 0.7–1.0 cm long), and the roots from the apical region were cut into segments of 0.7–1.0 cm length. Nine explants were placed in 50 ml of callus induction medium (CIM) in a 100 × 25 mm crystal-grade polystyrene Petri dish (Fisherbrand™) with 15 replications. The leaves and roots were placed separately, and the leaf explants were placed with the abaxial side down.

**Figure 1:**
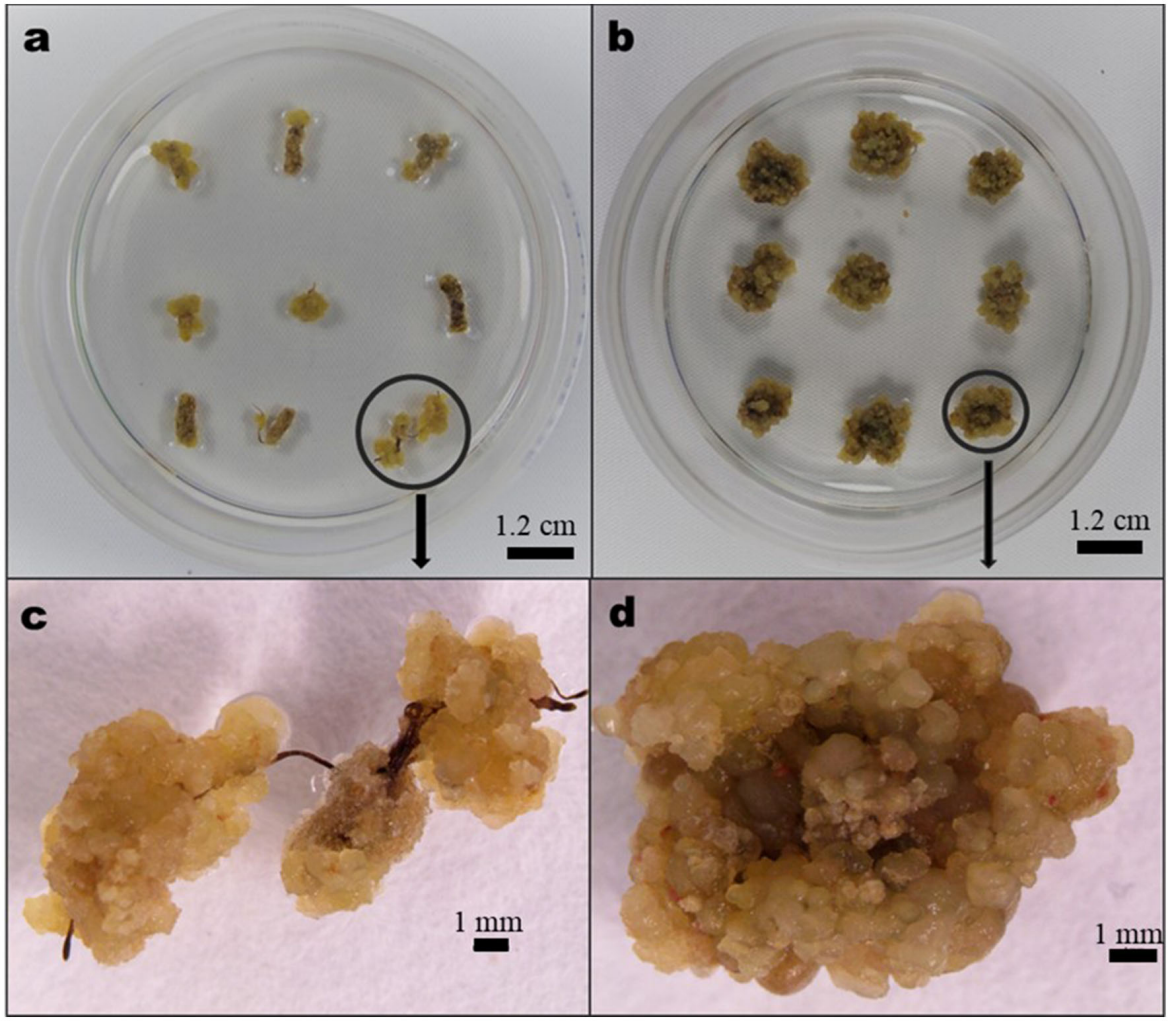
Characteristic nature of *Aspilia africana* callus: **(a)** calli from root explant generated in 0.5 mg/L benzylaminopurine (BAP) in combination with 1.0 mg/L 2,4-dichlorophenoxyacetic acid (2,4-D), **(b)** calli from leaf explant generated in 1.0 mg/L BAP combined with 1.0 mg/L 2,4-D, **(c)** magnified callus (encircled) in **(a)**, and **(d)** magnified callus (encircled) in **(b)**.

The MS medium supplemented with 1.0 mg/L of cytokinins [6-furfurylaminopurine, kinetin (KN), or benzylaminopurine (BAP)] either singly or in combination with 0.5 or 1.0 mg/L of auxin [1-naphthaleneacetic acid (NAA) or 2,4-dichlorophenoxyacetic acid (2,4-D)] or 1.0 mg/L of auxin (2,4-D or NAA) either singly or in combination with 0.5 mg/L KN or BAP was used as the CIM. The explants in CIM were kept at 25 ± 1°C and 70% relative humidity in the dark until the callus developed. Callus on each explant was considered formed when 25% of the cut surfaces of segments formed calli (Figure 1). The nature of the callus and the callus percentage induction were determined after 6 weeks of incubation.

### Regeneration of Shoots

**Figure 2:**
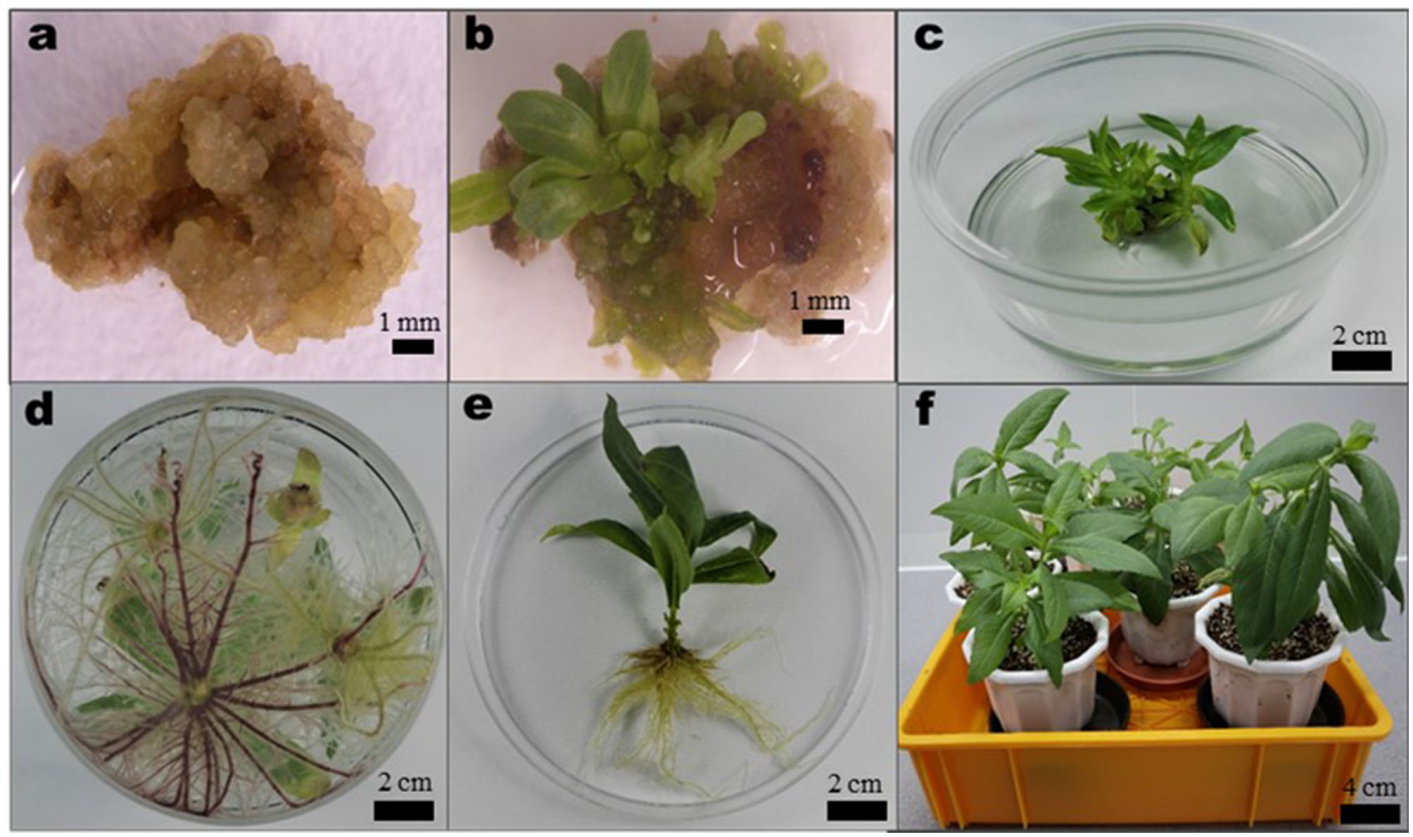
Summary of *in vitro* propagation of *A. africana* from callus. **(a)** Newly formed callus placed in the Murashige and Skoog (MS) medium supplemented with 1.0 mg/L BAP, 0.5 mg/L Thidiazuron (TDZ), and 0.05 mg/L 1-Naphthaleneacetic acid (NAA), **(b)** Shoot organogenesis occurring on callus after 7 weeks in regeneration medium, **(c)** developed shoots from callus, **(d)** rooting of *in vitro* regenerated shoots in 1/2 MS medium supplemented with 0.1 mg/L NAA, **(e)** fully developed plantlet removed from medium and roots washed, and **(f)** acclimatized potted plants.

Callus was moved to MS media augmented with auxin NAA (0.05, 0.1, 0.5, and 1.0 mg/L) and cytokinins [Thidiazuron (TDZ) or BAP] at various concentrations (0.5, 1.0, 1.5, 2.0, 2.5, and 3.0 mg/L) either singly or in combination with shoot regeneration. Ten replicates were made for each treatment, comprising six callus pieces (~0.8 cm^3^ per piece) in 50 ml of the shoot regeneration medium in a Petri dish (100 × 25 mm, Fisherbrand™). The callus cultures were kept at 25 ± 1°C and relative humidity of 70% under white fluorescent tubes (33.73 μmol/m^2^/s light intensity) in a 16-h photoperiod system. At 1-month intervals, until shoots regenerated, the *A. africana* calli (Figure 2a) were subcultured to fresh media of the same composition (Figures 2b,c). Shoots produced per callus were counted, and shoot regeneration (%) was determined. Regenerated *A. africana* shoots thereafter were moved to the media for rooting (Figure 2d).

### Rooting of Regenerated Shoots

The regenerated *A. africana* shoots were cultured in the MS medium (100 ml) of various strengths (i.e., 2 MS, MS, 1/2 MS, and 1/4 MS) supplemented with 0.1 mg/L NAA in polystyrene culture vessels (125 × 110 mm). Four regenerated *A. africana* shoots were set up in each culture vessel, with 20 replications for this experiment. Rooting rates, root lengths, and root numbers were determined for each treatment after culturing for 6 weeks. In our previous study, the auxin NAA at a concentration of 0.1 mg/L had been determined to be best for the rooting of *A. africana* shoots (Okello et al., 2021c). In this study, we were interested in determining which medium strength in combination with 0.1 mg/L NAA would produce optimum rooting results for the regenerated *A. africana* shoots.

### Acclimatization

After removing the media traces from the roots of each regenerated *A. africana* plantlet (Figure 2e) by rinsing in running water from a tap, the plants were moved to horticulture soil mixed with perlite (2:1 ratio) in the 22 cm diameter plastic pots (Figure 2f). The plants were covered with transparent plastic bags ensuring adequate humidity and kept in growth chambers operating under a 16-h photoperiod (33.73 μmol/m^2^/s light intensity) at ~25°C and 70% relative humidity. The polythene coverings were opened gradually after 2 weeks as the plantlets acclimatized. Plant survival rates were determined at 6 weeks following acclimatization.

### Histological Analysis

The histological analysis was performed on *A. africana* samples at different developmental stages (i.e., callus, the initial stage of shoot organogenesis, and completely developed plantlets). The tissues of the plant were washed to remove the traces of plant media and then successively dehydrated in 50, 70, 80, 90, 95, 98, and 100% ethanol (1 h in each) at 25 ± 1°C. After dehydration, the tissues were cleared in different proportions of xylene/ethanol mixtures (i.e., 1/4 xylene:3/4 ethanol; 1/2 xylene:1/2 ethanol; and 3/4 xylene:1/4 ethanol) and two times in xylene for 30 min in each clearing agent at 25 ± 2°C and then successively in paraffin for 1 h in each of the following: 1/3 paraffin:2/3 xylene; 2/3 paraffin:1/3 xylene; and two times in paraffin at 60°C, and finally fixed overnight in paraffin. The blocks of paraffin with the tissues were cut into 12-μm slices with a microtome, deparaffinized, and then successively rehydrated (5 min in each of the following): xylene (two times), 50% xylene, 50% ethanol, two times in 100% ethanol, 95% ethanol, 70% ethanol, and 50% ethanol. The rehydrated tissues were then stained as follows: 1% safranine (1 h), rinsed in water; 50%, 70%, and 95% ethanol successively (3 min in each); 3 min in fast-green; 100% ethanol (two times and 1 min each time); 30 min in carbol xylene (30 min); and xylene (three times for 5, 15, and 15 min, respectively). The *A. africana* plant tissue samples were taken with a light microscope (Olympus BX-53, Tokyo, Japan) and digital camera (Olympus DP21, Olympus, Tokyo, Japan) on mounting with Balsam.

### Statistical Analysis

All experimental data were analyzed by the one-way ANOVA with Tukey's *post-hoc* or Bonferroni's multiple comparison tests, using Prism (GraphPad software, version 5.03). In CIM, 9 leaf/root segment explants with 15 replications were used; for shoot regeneration, 6 callus pieces were used with 10 replications, and for rooting, 4 regenerated *A. africana* shoots with 20 replications were used. Means were regarded statistically significant at *p* ≤ 0.05.

## Results

### Seed Preparation and Germination

The sterilization process of the seeds was quite difficult, with up to 53% of the inoculated seeds of *A. africana* being contaminated. The *in vitro* germination percentage of *A. africana* seeds was fairly low (~55%) in the culture medium used. Seed germination was quick, averaging about 4 days.

### Callus Induction

The highest percentage of induced callus from *A. africana* leaf explants was 91.9 ± 2.96% in the MS medium augmented with 1.0 mg/L BAP together with 1.0 mg/L 2,4-D, although this did not significantly differ from those in media containing 1.0 mg/L BAP and 1.0 mg/L NAA (90.4 ± 2.84%); 1.0 mg/L BAP and 0.5 mg/L NAA (88.2 ± 3.51%); and 0.5 mg/L BAP and 0.5 mg/L NAA (88.1 ± 3.16%) among others (Table 1). The highest percentage of callus induction from the root explants was 92.6 ± 2.80% in the MS medium augmented with 0.5 mg/L BAP and 1.0 mg/L 2,4-D, followed by 90.4 ± 3.04% (in medium with 1.0 mg/L BAP and 1.0 mg/L 2,4-D); 87.4 ± 3.57% (in medium with 1.0 mg/L KN and 1.0 mg/L 2,4-D); then 75.6 ± 4.09% (in medium with 1.0 mg/L BAP and 0.5 mg/L 2,4-D), which were all significantly higher (*p* ≤ 0.05) than the percentage of callus induced in the rest of the treatments (Table 1). Among the treatments, the lowest percentage of callus induction was recorded in the explants cultivated on the MS medium augmented with 1.0 mg/L KN for both leaf (18.5 ± 4.81%) and root (00.0 ± 0.00%) explants. Generally, the medium supplemented with a combination of auxin and cytokinin had a better callus induction response than when auxins or cytokinins were used alone (Table 1). The MS medium without plant growth regulators (PGRs) did not produce any callus. The calli produced from both root and leaf explants were brown, cream, or pale yellow, and their textures were either compact or friable depending on the medium composition and explant type (Table 1, Figure 1).

**Table 1:** Percentage induction and characteristics of callus from leaf and root explants of *Aspilia africana*.

**Plant growth regulators (mg/L)**	**Leaf explants**			**Root explants**		
	**% Induction**	**Nature/Color**	**Texture**	**% Induction**	**Nature/Color**	**Texture**
BAP 1.0	26.7 ± 5.29^c^	Dark brown	Friable	10.4 ± 3.83^fg^	Dark brown	Friable
BAP 0.5: NAA 1.0	88.1 ± 3.16^a^	Cream	Friable	59.3 ± 3.86^cd^	Cream	Compact
BAP 1.0: NAA 1.0	90.4 ± 2.84^a^	Cream	Compact	63.0 ± 5.39^cd^	Pale yellow	Compact
BAP1.0: NAA 0.5	88.2 ± 3.51^a^	Brown	Friable	43.7 ± 3.67^de^	Pale green	Compact
NAA 1.0	29.6 ± 5.39^c^	Brown	Friable	13.3 ± 3.79^fg^	Cream	Friable
BAP 0.5: 2,4-D 1.0	87.4 ± 3.74^a^	Pale green	Compact	92.6 ± 2.80^a^	Pale yellow	Compact
BAP 1.0: 2,4-D 1.0	91.9 ± 2.96^a^	Pale green	Compact	90.4 ± 3.04^a^	Pale yellow	Compact
BAP 1.0: 2,4-D 0.5	85.9 ± 3.51^a^	Dark green	Compact	75.6 ± 4.09^abc^	Pale yellow	Compact
2,4-D 1.0	52.6 ± 6.37^b^	Dark green	Compact	33.3 ± 5.74^ef^	Pale yellow	Compact
KN 0.5: 2,4-D 1.0	73.3 ± 5.18^ab^	Dark green	Compact	60.0 ± 5.82^cd^	Yellow	Compact
KN 1.0: 2,4-D 1.0	85.2 ± 5.91^a^	Dark green	Compact	87.4 ± 3.57^ab^	Pale green	Friable
KN 1.0: 2,4-D 0.5	83.7 ± 3.23^a^	Brown	Friable	67.4 ± 4.26^bc^	Brown	Friable
KN 1.0	18.5 ± 4.81^c^	Brown	Friable	0.0 ± 0.00^g^		
KN 0.5: NAA 1.0	39.3 ± 5.72^bc^	Cream	Friable	11.1 ± 3.25^fg^	Cream	Friable
KN 1.0: NAA 1.0	67.4 ± 4.66^ab^	Cream	Friable	21.5 ± 5.02^f^	Brown	Friable
KN 1.0: NAA 0.5	65.2 ± 3.57^ab^	Dark brown	Friable	20.7 ± 4.18^f^	Cream	Friable
MS	0.0 ± 0.00^d^	Cream	Friable	0.0 ± 0.00^g^		

*Means (± standard error) within a column followed by same superscript letter are not significantly different using Tukey's multiple comparison test and p = 0.05*.

### Shoot Regeneration

**Figure 3:**
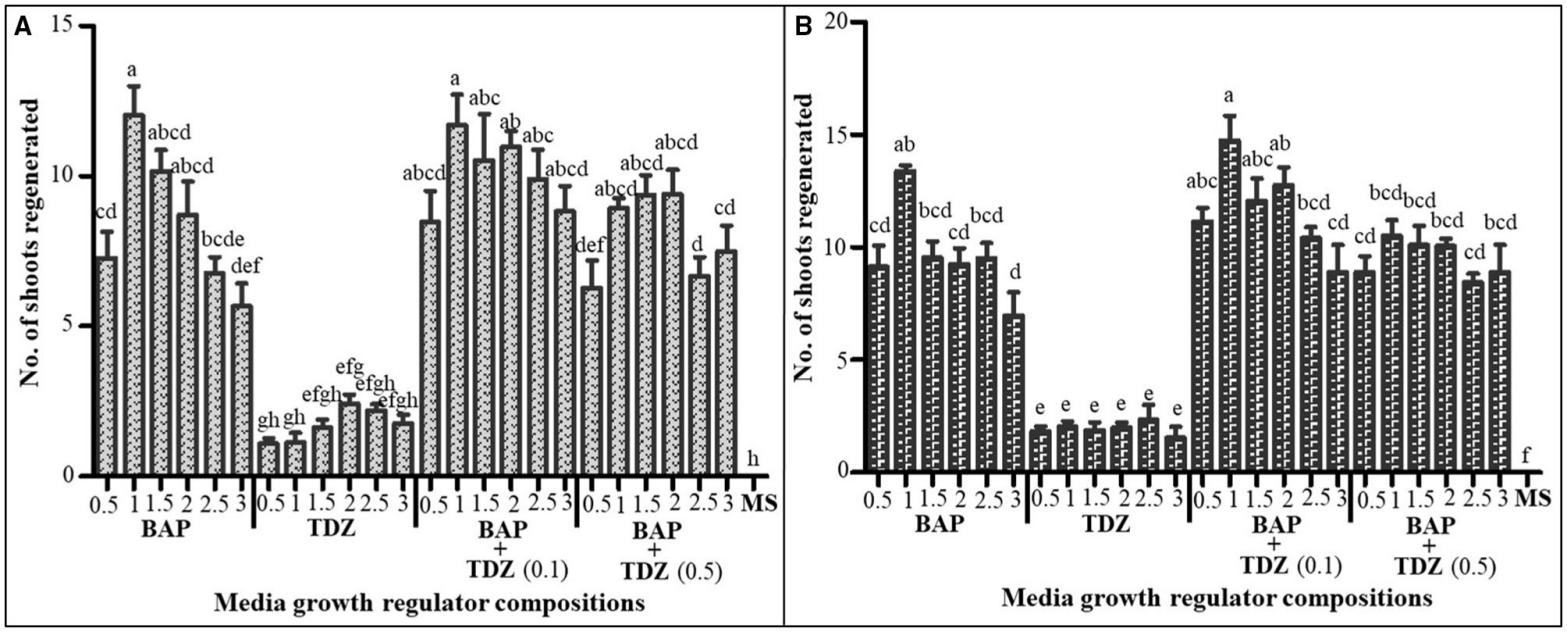
The number of shoots regenerated from calli in MS medium supplemented with different plant growth regulator combinations **(A)** from leaf explant calli and **(B)** from root explant calli. All media contained 0.05 mg/L NAA, and all concentrations are in mg/L. Values are presented as means ± SE. The same letters are not significantly different by Tukey's multiple comparison test and *p* = 0.05.

For the leaf explants, we found that the MS medium augmented with 1.0 mg/L BAP and 0.05 mg/L NAA had the highest regeneration percentage of shoots (80.0 ± 6.23%) and the highest shoot number per callus (12.0 ± 6.23); this was followed by the medium supplemented with 0.1 mg/L TDZ in addition to 1.0 mg/L BAP and 0.05 mg/L NAA (76.7 ± 4.08% shoot regeneration percentage; 11.70 ± 1.01 number of shoots per callus) (Table 2, Figure 3A). Meanwhile, for the root explants, the highest percentage of regeneration (86.7 ± 6.24%) and the number of shoots per callus (14.7 ± 1.11) were recorded in the explants cultivated on the MS medium augmented with 1.0 mg/L BAP, 0.05 mg/L NAA, and 0.1 mg/L TDZ, followed by the results in the medium with the same composition but without TDZ (with shoot regeneration percentage and the number of shoots per callus, 83.3 ± 5.27% and 13.4 ± 0.28, respectively) (Table 2, Figure 3B).

**Table 2:** Percentage shoot regeneration from calli obtained from leaf and root explants of *A. africana*.

**Plant Growth Regulators (mg/L)**				**Percentage shoot regeneration**	
				**Leaf explants**	**Root explants**
BAP	0.5			60.0 ± 8.50 ^abcde^	66.7 ± 7.45 ^abcde^
	1.0			80.0 ± 6.23 ^a^	83.3 ± 5.27 ^ab^
	1.5			73.3 ± 4.08 ^abcd^	70.0 ± 6.23 ^abcd^
	2.0			70.0 ± 6.24 ^abcd^	63.3 ± 6.24 ^abcd^
	2.5			63.7 ± 6.23 ^abcde^	66.7 ± 5.27 ^abcd^
	3.0			46.7 ± 6.25 ^bcde^	50.0 ± 7.45 ^bcd^
TDZ	0.5			27.3 ± 7.99 ^fg^	33.3 ± 9.13 ^e^
	1.0			26.7 ± 6.67 ^fg^	40.0 ± 4.08 ^cde^
	1.5			33.3 ± 5.27 ^ef^	36.7 ± 9.72 ^de^
	2.0			46.7 ± 6.24 ^cdef^	33.3 ± 5.27 ^e^
	2.5			40.0 ± 4.08 ^def^	36.7 ± 9.72 ^de^
	3.0			30.0 ± 6.23 ^f^	23.3 ± 6.67 ^ef^
BAP	0.5	TDZ	0.1	66.7 ± 7.45 ^abcd^	73.3 ± 4.08 ^abc^
	1.0		0.1	76.7 ± 4.08 ^abc^	86.7 ± 6.24 ^a^
	1.5		0.1	66.7 ± 7.45 ^abcd^	70.0 ± 6.24 ^abcd^
	2.0		0.1	63.3 ± 3.33 ^abcde^	80.0 ± 6.23 ^ab^
	2.5		0.1	60.0 ± 4.08 ^abcde^	73.3 ± 4.08 ^abc^
	3.0		0.1	56.7 ± 8.49 ^abcdef^	63.3 ± 9.72 ^abcde^
BAP	0.5	TDZ	0.5	50.0 ± 9.13 ^abcdef^	63.3 ± 8.16 ^abcde^
	1.0		0.5	73.3 ± 4.08 ^abcd^	70.0 ± 6.24 ^abcd^
	1.5		0.5	53.3 ± 6.23 ^abcdef^	70.0 ± 6.24 ^abcd^
	2.0		0.5	63.3 ± 3.33 ^abcde^	73.3 ± 4.08 ^abc^
	2.5		0.5	53.3 ± 6.24 ^abcdef^	60.0 ± 4.08 ^abcde^
	3.0		0.5	53.3 ± 3.33 ^abcdef^	63.3 ± 9.71 ^abcde^
MS				0.0 ± 0.00 ^g^	0.0 ± 0.00 ^g^

*All media except MS contained 0.05 mg/L NAA. Means (± standard error) within a column followed by same superscript letter are not significantly different using Tukey's multiple comparison test and p= 0.05*.

Both shoot regeneration percentage and shoot number per callus were generally higher in media supplemented with two cytokinins (i.e., BAP and TDZ) compared to when only a single cytokinin was used in combination with auxin (NAA) (Table 2, Figure 3), although the differences were not statistically significant. Media supplemented with TDZ in combination with NAA were generally associated with low shoot regeneration percentage and a low number of shoots for all calli (Table 2, Figure 3).

Only the combinations of cytokinins with 0.05 mg/L NAA are presented in Figure 3 and Table 2 because all other combinations produced no shoots but only roots.

### Rooting

**Figure 4:**
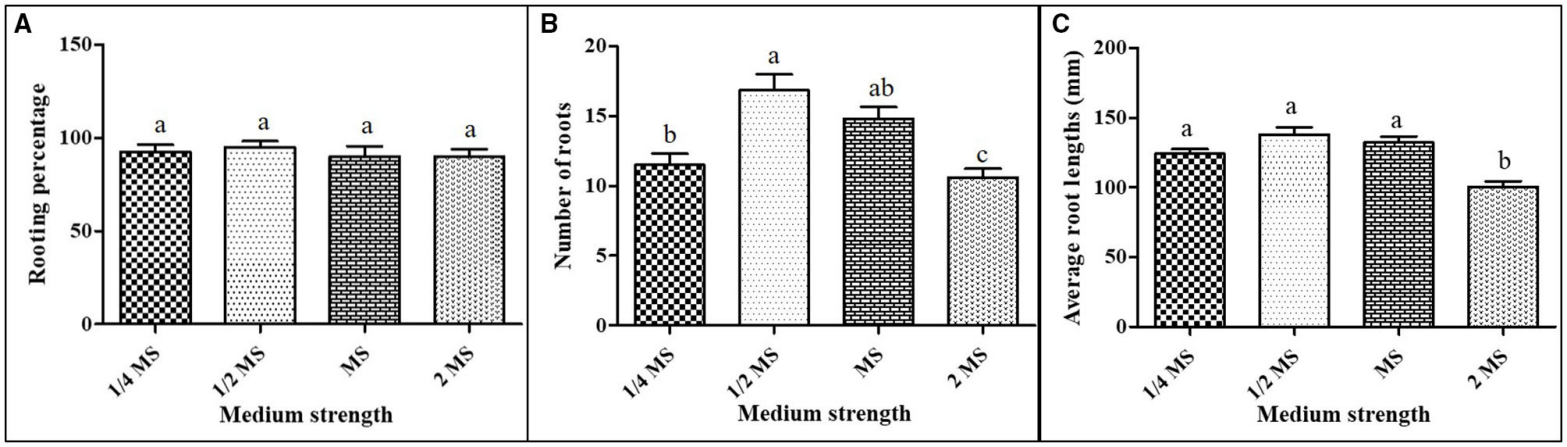
Rooting of *A. africana* regenerated shoots in MS media of different strengths supplemented with 0.1 mg/L NAA: **(A)** the number of roots, **(B)** root lengths, and **(C)** rooting percentage. Values are presented as means ± SE. The same letters are not significantly different by Tukey's multiple comparison test and *p* = 0.05.

The rooting of micropropagated *A. africana* shoots had great success, with over 90% rooting in all treatments (Figure 4A). Although 1/2 MS medium supplemented with 0.1 mg/L NAA had the highest rooting percentage at 95.0 ± 3.33%, it did not vary significantly from the rooting percentage in the rest of the treatments (Figure 4A). The highest average number of roots (16.85 ± 3.33) was recorded in 1/2 MS supplemented with 0.1 mg/L NAA (Figure 4B). It differed significantly (*p* ≤ 0.05) from average root lengths of both 1/4 MS and 2 MS augmented with 0.1 mg/L NAA but not MS with 0.1 mg/L NAA (Figure 4B). Average root lengths of the micropropagated *A. africana* plants in 1/4 MS, 1/2 MS, and MS all supplemented with 0.1 mg/L NAA did not vary significantly (*p* ≤ 0.05) although the longest average root length (138.1 ± 3.33 mm) was recorded in 1/2 MS augmented with 0.1 mg/L NAA (Figure 4C).

### Acclimatization

After 6 weeks of acclimatization, the rooted *A. africana in vitro* regenerated plants had a high survival rate (93.6%). The acclimatized *A. africana* plants grew well and displayed normal growth characteristics and morphology typical of the plant species.

### Histological Analysis

**Figure 5:**
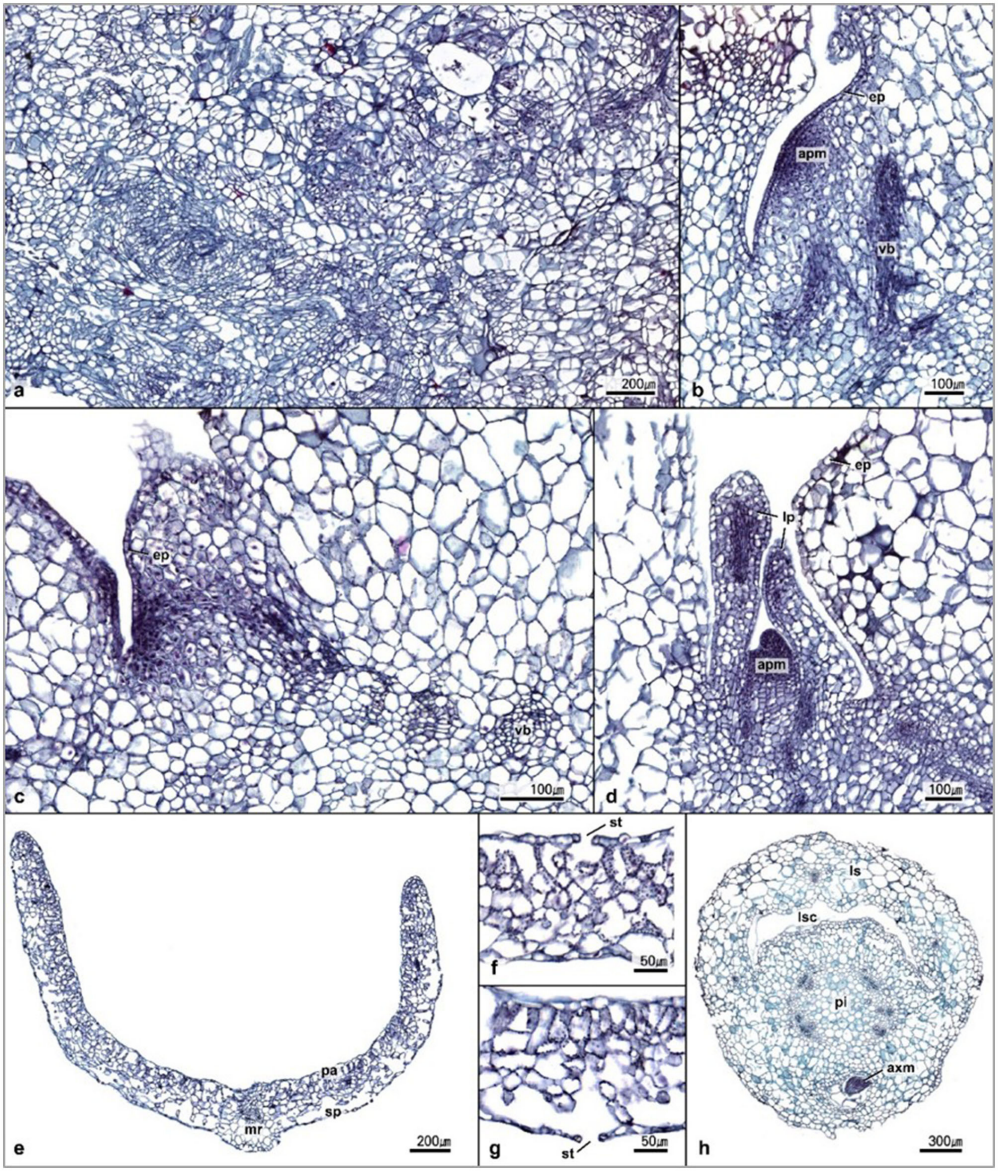
Transverse sections of *A. africana*. **(a)** The anatomical structure of callus, **(b,c)** initiation of shoot apical meristem, **(d)** shoot apical meristem and leaf primordial, **(e–g)** transverse section of young leaf, and **(h)** transverse section of young stem. apm, apical meristem; ep, epidermis; vb, vascular bundle; mr, midrib; pa, palisade parenchyma; sp, sponge parenchyma; st, stomata; lp, leaf primordial; ls, leaf sheath; lsc, leaf sheath canal; pi, pith; axm, axillary meristem.

The callus was mainly composed of isodiametric, small-sized, and compact parenchymal cells with dense cytoplasm and signs of early prevascular development (Figure 5a). Early prevascular development was irregular, without directionality or axiality.

As vascular development progressed, the vascular bundle exhibited constant axiality. At the earlier stage of organogenesis, the developing shoot apical meristems were evident, and well-developed shoot buds could be observed through the section (Figures 5b–d). The tissues at the stage of differentiation consisted of a dense network of prevascular bundles (Figures 5b–d). Initial cells in the mature epidermis and shoot meristems were observed, of which, more mature ones in shoot meristems formed the apical meristem and leaf primordia (Figures 5b–d). The differentiated shoot contained prominent leaf primordium with protective layers of tissue around the apical meristem, which was dome-shaped (Figure 5d).

Sections through the fully developed plantlet tissues clearly showed differentiated tissues in the leaf, stem, and well-developed organs with prominent phloem and xylem tissues (Figures 5e–h). Transverse sections through the leaf tissue showed a high concentration of chloroplasts at the adaxial side and considerably fewer on the abaxial side (Figures 5e–g). The palisade and spongy parenchyma cells were distributed on the adaxial and abaxial surfaces of young leaves, respectively, and stomatal cells were found on both surfaces (Figures 5e–g). The epidermal and parenchymatous cells of the leaf tissue were poorly differentiated and irregularly arranged with mesophyll cells having very large intercellular airspaces (Figures 5e–g). The basal part of the petiole became a sheath that covered the stem, leaving a broad leaf-sheath canal (Figure 5h). The stem tissue was very well-differentiated, with organized and prominent vascular bundles (Figure 5h).

## Discussion

The exogenous application of cytokinins and auxins is known to induce callus formation in plants (Ikeuchi et al., 2013). We observed that the rate of callus induction from both leaf and root explants was significantly higher (*p* < 0.05) in media supplemented with the combinations of cytokinins and auxins than in media containing either cytokinins or auxins alone. Similar observations have been made for many other plant species in the same family as *A. africana*, such as *Elephantopus scaber* (Abraham and Thomas, 2015), *Centaurea arifolia* (Yüzbaşioglu et al., 2012), *Stevia rebaudiana* (Gupta et al., 2010; Narender et al., 2011; El-Zaidy et al., 2014), *Senecio candicans* (Hariprasath et al., 2015), and *Pentanema indicum* (Sivanesan and Jeong, 2007), indicating a synergistic effect of cytokinin-auxin interaction in callus induction (Chirumamilla et al., 2021).

Several studies have demonstrated that root and leaf explants could be successfully used for calli induction and subsequent regeneration of plants (Dale and Deambrogio, 1979; Franklin et al., 2004; Hoque and Mansfield, 2004; Ahmad et al., 2010; Kumar et al., 2013). Root and shoot explants inoculated on cytokinin- and auxin-containing CIM generates calli from pericycle cells adjacent to xylem poles, as observed in *Arabidopsis* (Atta et al., 2009). *In vitro* propagation studies of some species of the Asteraceae family, such as *Carthamus tinctorius* (Ghasempour et al., 2014) and *Stevia rebaudiana* (Gupta et al., 2010), where the potential for callus induction in different explants was compared, showed that the callus induction rate from leaf explants was generally higher than that from root explants. The rate of callus induction is dependent on the type of explant used, its developmental stage, and its genotype (Holme and Petersen, 1996). The variation in the levels of endogenous growth regulators, such as cytokinins, auxins, and abscisic acid, in different plant tissues could cause different response levels to callus induction in the different explants (Holme and Petersen, 1996).

Only regeneration-competent cells can form callus in plant tissues (Che et al., 2007; Liu et al., 2014), and in *Arabidopsis* leaves, some vascular parenchyma cells and procambium serve as regeneration-competent cells, whereas in the roots, it is the xylem-pole pericycle cells that form callus (Guo et al., 2018). Thus, the variation in the differentiation status of the regeneration-competent cells in the different explants may also contribute to different callus induction responses in leaf and root explants of *A. africana* (Guo et al., 2018). Calli can be categorized, depending on their macroscopic features, as compact or friable if they do not possess apparent organ regeneration, or if they do show some tendency for organ regeneration, they can be categorized as rooty, shooty, or embryonic, depending on the organ the callus is tending to regenerate (Guo et al., 2018). In our case, all the calli formed from both root and shoot explants of *A. africana* were either friable or compact but varied in color and nature depending on the explant and media/hormonal combinations.

Although supplementing plant tissue culture media with cytokinins and auxins promotes callus induction, the callus induction rate depends on, among other factors, the effect of synergistic interactions between different classes of growth regulators that result in dedifferentiation (Cordeiro et al., 2007; Santos et al., 2014). All media combinations of BAP with 2,4-D had very high callus induction responses for both leaf and root explants with the best being a combination of 1.0 mg/L BAP and 1.0 mg/L 2,4-D for leaf explants and 0.5 mg/L BAP with 1.0 mg/L 2,4-D for root explants. Similar observations have also been made in many studies on various species such as *Solanum khasianum* (Chirumamilla et al., 2021), *Gynura procumbens* (Nurokhman et al., 2019), *Trachyspermum ammi* (Fazeli-Nasab, 2018), *Tridax procumbens* (Wani et al., 2010), *Stevia rebaudiana* (Ahmad et al., 2011), and *Chrysanthemum morifolium* (Khan et al., 2020).

The auxin 2,4-D is known in plant micropropagation work to be suitable for callus induction in most plant species (Tahir et al., 2011). It initiates division of cells and controls cell growth as well as cell elongation (Santos et al., 2014), and according to previous studies, its combination with BAP gave the greatest synergistic effects as far as callus induction was concerned (Ahmad et al., 2011; Nurokhman et al., 2019). In our study, although a combination of BAP and NAA gave high callus induction responses from leaf explants, as previously recorded in other members of the same plant family (Asteraceae), such as *S. rebaudiana* (Patel and Shah, 2009), *Aster scaber* (Boo et al., 2015), and *Cichorium pumilum* (Al Khateeb et al., 2012), the response was much lower in root explants, confirming previous observations that explants obtained from different plant organs respond differently to callus induction treatment (Gupta et al., 2010; Ghasempour et al., 2014).

In this study, we observed that the MS plant culture medium supplemented with 1.0 mg/L BAP and 0.05 mg/L NAA was very effective to induce shoot regeneration from calli from both root and leaf explants of *A. africana*. The presence of cytokinins and auxins is essential for callus differentiation and regeneration in plants (Kim et al., 2021). In fact, a combination of NAA and BAP has been shown to induce high shoot organogenic potential in calli of many species in the family Asteraceae such as *Echinacea purpurea* (Koroch et al., 2002), *E. scaber* (Abraham and Thomas, 2015), *Dendranthema grandiflorum* (Khandakar et al., 2014), *Carthamus tinctorius* (Ghasempour et al., 2014), and *Eclipta alba* (Sharma et al., 2013).

High concentrations of cytokinins have negative effects on shoot regeneration (Wang et al., 2018). We observed in our study that lower concentrations of BAP (2.0 mg/L or less) had the highest probability of regenerating shoots from all calli, with regeneration percentage and number of formed shoots greater than in higher concentrations (>2.0 mg/L). Rasool et al. (2009) noted that greater concentrations of BAP tend to retard shoot multiplication and growth, as the concentrations may be beyond the optimum levels for the plants. Similar observations have been made for many other species such as *E. purpurea* (Koroch et al., 2002), *E. scaber* (Abraham and Thomas, 2015), *D. grandiflorum* (Khandakar et al., 2014), and *C. tinctorius* (Ghasempour et al., 2014), although contrary to these findings, shoot regeneration from callus in a few species within the Asteraceae family was favored by high concentrations of BAP, for instance, in 5.0 mg/L BAP in *S. rebaudiana* (Sairkar et al., 2009). These observations indicate that plant species respond differently to varying PGR concentrations. Interestingly, in our study, all media with the concentrations of NAA higher than 0.1 mg/L resulted only in root organogenesis irrespective of the cytokinin used, which was not the case in previous studies among other species, where concentrations higher than 0.1 mg/L formed shoots on supplementation with BAP (Sairkar et al., 2009; Rout and Sahoo, 2013). Koroch et al. (2002) observed that an increased concentration of NAA resulted in less shoot organogenesis and more callus multiplication. Regeneration responses to PGRs vary depending on the species, as observed by Kim et al. (2021).

We also observed that the explants cultivated on media supplemented with only NAA and TDZ had a very low percentage of shoot regeneration and a low number of regenerated shoots, but the addition of BAP to the media increased shoot regeneration capacities of the calli. This demonstrates a synergistic interaction that increases shoot regeneration from callus. It is well-known that a synergistic effect between different cytokinins results in high regeneration rates (Wang et al., 2018). Abdolinejad et al. (2020) reported similar findings, where a medium augmented with BA, NAA, and TDZ greatly enhanced shoot regeneration and growth from callus. Furthermore, they observed that BAP, TDZ, and NAA at relatively high, moderate, and low concentrations, respectively, converted morphogenic calli into bud primordia that developed into shoots.

In our previous study, we noted that for the rooting of *A. africana* regenerated plantlets, 0.1 mg/L NAA performed best in terms of rooting percentage, root length, and root numbers compared to all its other concentrations and indole-3-butyric acid (IBA) and indole-3-acetic acid (IAA) (Okello et al., 2021c). Therefore, in this study, we were interested in determining the media strength in which micropropagated *A. africana* plants would best perform when augmented with 0.1 mg/L NAA. Rooting responses for all treatments were very good, with all having a very high rooting percentage (>90) and more than 10 roots per plantlet, with average lengths all above 100 mm (Figure 4C). This high rooting response could be attributed to the ideal concentration of auxin NAA, which greatly boosted the rooting of regenerated plantlets. Auxins play a crucial role in root initiation and growth (Okello et al., 2021c).

The auxin NAA has been reported to induce maximum rooting in many other plants, such as *S. rebaudiana* (Rafiq et al., 2007), *Lychnophora pinaster* (De Souza et al., 2007), and *D. grandiflorum* (Khandakar et al., 2014). The auxin NAA at 0.1 mg/L concentration has also been demonstrated to be best for the rooting of micropropagated shoots of some members of the Asteraceae family, for instance, *S. rebaudiana* (Ahmed et al. (2007) and *D. grandiflorum* (Khandakar et al., 2014). High concentrations of NAA have been reported to inhibit the root growth of plants, as was earlier observed in *A. africana* (Okello et al., 2021c) and *Arabidopsis* (Ivanchenko et al., 2010).

In this study, although all rooting responses were very good, the best, with consistently highest values of all the measured rooting parameters, was in 1/2 MS augmented with 0.1 mg/L NAA, although were not significantly different from other treatments for some parameters. In agreement with our findings, a half-strength MS medium was reported in several studies to be effective for the rooting of many *in vitro* regenerated plant species such as *Ruscus hypophyllum* (Winarto and Setyawati, 2014), *Phlox Pilosa* (Chen et al., 2020), and *Phellodendron amurense* (Yang et al., 2011). Hariprasath et al. (2015) also recorded optimum rooting in *S. candicans* in 1/2 MS medium, although it was supplemented with a much greater concentration of 3.0 mg/L NAA. In a different study, Abraham and Thomas (2015) noted the best rooting response in *E. scaber* but when the 1/2 MS was supplemented with IBA.

*In vitro* regenerated plants show some form of atypical anatomy, morphology, and physiology and, therefore, need time to acclimatize, as this is essential for their successive field survival (Komakech et al., 2020; Okello et al., 2021c). In this study, we noted that the micropropagated *A. africana* plants had a high rate of survival (93.6%) at acclimatization, which is close to the survival rate of 95.7% recorded for the same species in a previous study (Okello et al., 2021c). It is quite common for plant species in the family Asteraceae to show high survival rates, as has been observed in *E. alba* (Singh et al., 2012), *S. rebaudiana* (Hwang, 2006), and *E. scaber* (Abraham and Thomas, 2016).

Interestingly, a study of the total phenolic, flavonoid, and chlorogenic acid contents of the *in vitro* regenerated callus and juvenile plant tissues showed high quantities of these pharmacologically important phytochemicals (Okello et al., 2021a). The biological activities of medicinal plants, such as antioxidant, antimicrobial, and anti-inflammatory activities, largely depend on these phytochemicals. We found that the *in vitro* regenerated tissues had high antioxidant activity (Okello et al., 2021a). These *in vitro* regenerated plant tissues could be a valuable natural source of antioxidants and could be further exploited for the development of useful pharmaceutical products (Okello et al., 2021a).

The presence of compact, small-sized, and isodiametric cells with dense cytoplasm cells in the callus tissue indicated that such regions of the callus tissue were embryogenic (Binte Mostafiz and Wagiran, 2018; Kim et al., 2021). According to the study by Vega et al. (2009), cells with dense cytoplasm and abundant starch granules are metabolically active and can undergo dedifferentiation, forming embryogenic calli.

Kim et al. (2021) observed that in *Asparagus cochinchinensis*, meristematic centers protruded to form dome-shaped apical meristems. In our study, the shoot apical meristems were covered by a protective layer of leaf primordia from differentiated meristems, as was previously observed for *Asparagus cochinchinensis* (Kim et al., 2021) and *Curcuma zedoaria* (Mello et al., 2001).

We observed, through the cross-sections of the stem, that the tissues of the developed plantlets were well-differentiated with fully developed vascular bundles and were functional. Kim et al. (2021) explained that the presence of well-differentiated tissues is an indication that the plant is self-sustaining through photosynthetic and respiratory activities as is important for their survival during acclimatization. A large number of chloroplasts were observed in the leaf tissues, indicating that the organ was photosynthetic (Okello et al., 2021). Similar to our observations, Okello et al. (2021c) also noted that the cross-section of a leaf of *in vitro* regenerated *A. africana* plants revealed less differentiated and unorganized cells with very large intercellular airspaces. Okello et al. (2021c) explained that such abnormal features occur as a result of the stress response of the plants to the *in vitro* culture conditions. In addition, Shekhawat and Manokari (2018) noted that leaf tissues of *in vitro* regenerated plants fully differentiate and develop upon transfer to the external environment during acclimatization.

## Conclusion

In this study, we reported that full-strength MS media augmented with either 1.0 mg/L BAP and 1.0 mg/L NAA or 1.0 mg/L BAP and 1.0 mg/L 2,4-D were the most effective for the induction of callus from leaf explants, with a callus induction percentage of over 90%. For the root explants, the medium supplemented with 1.0 mg/L 2,4-D and 0.5 mg/L BAP was most ideal for callus induction (at a percentage of 92.6 ± 2.80%), and the MS medium augmented with 1.0 mg/L BAP, 0.05 mg/L NAA, and 0.1 mg/L TDZ was best for the percentage regeneration (86.7 ± 6.24%) and the number of shoots per callus (14.7 ± 1.11). For the leaf-derived calli, MS plant tissue culture medium supplemented with 1.0 mg/L BAP and 0.05 mg/L NAA was best for the shoot regeneration (at a percentage of 80.0 ± 6.23%) and the number of shoots per callus (12.0 ± 6.23). The regenerated *A. africana* plantlets developed efficient root systems with the highest rooting percentage (95.0 ± 3.33%), the highest number of roots (16.85 ± 3.33), and the longest root length (138.1 ± 3.33 mm) in 1/2 MS plant culture medium augmented with 0.1 mg/L NAA. During acclimatization, the regenerated plants survived at a rate of 93.6%. The tissue of the stem was fully differentiated with prominent vascular bundles, comprising the xylem and phloem tissues, and had clear leaf-sheath canals and axillary meristems, while the young leaf tissue consisted of cells that were largely unorganized and poorly differentiated cells with large intercellular airspaces typical of *in vitro* regenerated young leaf tissues. The leaf tissues of the micropropagated plants differentiated fully during acclimatization (Shekhawat and Manokari, 2018; Okello et al., 2021c). The *in vitro* regenerated tissues were shown to contain high total flavonoids, phenolics, and chlorogenic acids; had good antioxidant potential; and could be used as a sustainable source of raw materials in the development of important pharmaceutical products (Okello et al., 2021a). Our study presents an efficient reproducible protocol for the indirect regeneration of *A. africana*, which offers a basis for its large-scale multiplication, domestication, and germplasm preservation. This is the first study to develop an indirect regeneration protocol for *A. africana* and to carry out the anatomical assessment through the different stages of development from callus to a fully developed plantlet.

## Data Availability Statement

The original contributions presented in the study are included in the article/supplementary material, further inquiries can be directed to the corresponding author/s.

## Author Contributions

DO conceived the research idea, designed the experimental plan, participated in every stage and all parts of the research work, performed the statistical analyses, and wrote the manuscript. RK participated in all the plant tissue culture experiments. SY did the histological analysis. RG collected the plant materials and wrote the manuscript. ER, YC, FO, and AL read and improved the manuscript. YK provided technical guidance, supervised the whole research work, and read and improved the manuscript. All the authors read and approved the final manuscript.

## Funding

This study was supported under the framework of the International Cooperation Program (Korea-South Africa Cooperative Research Project for Excavation of Candidate Resources of Complementary and Alternative Medicine) managed by the National Research Foundation of Korea (Grant No. 2017093655 and KIOM: D17470). This study was also supported by the Development of Sustainable Application for Standard Herbal Resources (KSN2013320), the Korea Institute of Oriental Medicine (KIOM) through the Ministry of Science and ICT, South Korea, and the UST Overseas Training Program 2021, funded by the University of Science and Technology, South Korea.

## Conflict of Interest

The authors declare that the research was conducted in the absence of any commercial or financial relationships that could be construed as a potential conflict of interest.

## Publisher's Note

All claims expressed in this article are solely those of the authors and do not necessarily represent those of their affiliated organizations, or those of the publisher, the editors and the reviewers. Any product that may be evaluated in this article, or claim that may be made by its manufacturer, is not guaranteed or endorsed by the publisher.
